# Management of vascular access inflow-outflow imbalance: A bimodal approach

**DOI:** 10.1177/11297298241272166

**Published:** 2024-09-19

**Authors:** Gerald A Beathard, William C Jennings, Jan Malik, Haimanot Wasse, Bart L Dolmatch, John Ross, Surendra Shenoy, Roy-Chaudhury Pabir, Bharat Sachdeva, Dheeraj Rajan, Vandana Dua Niyyar, George M Nassar, Eric Peden, Timmy Lee, Gordon McLennan, Robert Shahverdyan

**Affiliations:** 1 Department of Medicine, University of Texas Medical Branch, Galveston, TX, USA; 2 Department of Surgery, University of Oklahoma College of Medicine, Tulsa, OK, USA; 3 Third Department of Internal Medicine, Charles University, Prague, Czech Republic; 4 Department of Medicine, Rush University Medical Center, Chicago, IL, USA; 5 Department of Radiology, Palo Alto Medical Foundation, Palo Alto, CA, USA; 6 Dialysis Access Institute, Orangeburg Regional Medical Center, Orangeburg, SC, USA; 7 Department of Surgery, Washington University School of Medicine, St. Louis, MO, USA; 8 Department of Medicine, University of North Carolina, Chapel Hill, NC, USA; 9 Department of Medicine, LSU Health Shreveport, Shreveport, LA, USA; 10 Department of Radiology, University of Toronto, Toronto, ON, Canada; 11 Department of Medicine, Emory University School of Medicine, Atlanta, GA, USA; 12 Department of Medicine, Weill Cornell Medical College, Houston, TX, USA; 13 Department of Cardiovascular Surgery, Weill Cornell Medical College, Houston, TX, USA; 14 Department of Medicine, University of Alabama, Birmingham, AL, USA; 15 Clinical Research, UC Health University of Colorado Hospital, Aurora, CO, USA; 16 Vascular Access Center, Asklepios Clinic Barmbek, Hamburg, Germany

**Keywords:** Dialysis access, techniques and procedures, interventional nephrology, cardiac complications of AV access, AV access flow reduction, excess AV access blood flow

## Abstract

A more accurate descriptive and clinically useful diagnosis based upon pathophysiology for what is commonly referred to as venous outflow stenosis is inflow-outflow imbalance. In these cases, the total outflow capacity of the AV access is inadequate to handle the inflow volume (Qa) without an increase in pressure. The relative inadequacy of the access outflow capacity in comparison to Qa results in increased outflow resistance and a proportional increase in intraluminal pressure. The clinical indicators associated with venous stenosis are the resulting manifestations of this imbalance. The point at which this occurs is dependent upon variations in these two parameters—Qa and outflow resistance. The variations in these two parameters are considerable and reciprocal. Excessive Qa results in or can lead to an entire list of serious problems that adversely affect patient morbidity and mortality. Most studies dealing with AV access Qa reduction have been for the treatment of an existing condition rather than its prevention; however, prevention of disease rather than waiting for its development is an important tenet of medical practice. The resulting clinical picture of inflow-outflow imbalance is taken as an indication for corrective treatment. In the past, in most cases this has meant angioplasty to open the outflow if it is reduced; however, this clinical picture may be associated with an excessive Qa and angioplasty in these cases creates the risk for a further increase in Qa. It is the authors’ opinion that access flow measurements should be a part of the evaluation of these cases prior to planning treatment. Using this information, a bimodal approach to primary treatment should be adopted involving either angioplasty for cases with a low or normal Qa or flow reduction in cases with an elevated Qa.

## Introduction

A common complication associated with the arteriovenous fistula (AVF) is venous stenosis affecting venous outflow. The KDOQI Clinical Practice Guidelines for Vascular Access^1^ (KDOQI Guidelines) state that a clinically significant stenosis, that is, critical stenosis, within the hemodialysis access circuit is associated with clinical indicators which can be determined either by physical examination of the arteriovenous (AV) access or the recognition of problems occurring during dialysis treatment (Table 1).

**Table 1.** Clinical indicators of AV access flow dysfunction.

Problems noted by physical examination
Hyperpulsatility of the access
Abnormal thrill—decreased, loss of diastolic component
Localized thrill along course of draining vein (site of stenosis)
Abnormal bruit—high pitched, loss of diastolic component
Failure of fistula to collapse when arm is elevated above level of heart
Development of aneurysms and pseudoaneurysms
Problems noted with dialysis treatment
Persistently increased venous pressure
Inability to achieve target dialysis blood flow rate (Qb)
Unexplained (>0.2 units) decrease in the delivered dialysis dose (Kt/V) with a constant dialysis
prescription and without prolongation of dialysis duration
Prolonged bleeding post needle removal, persistent
Evidence of recirculation
Thrombosis of access

**Figure 1.**
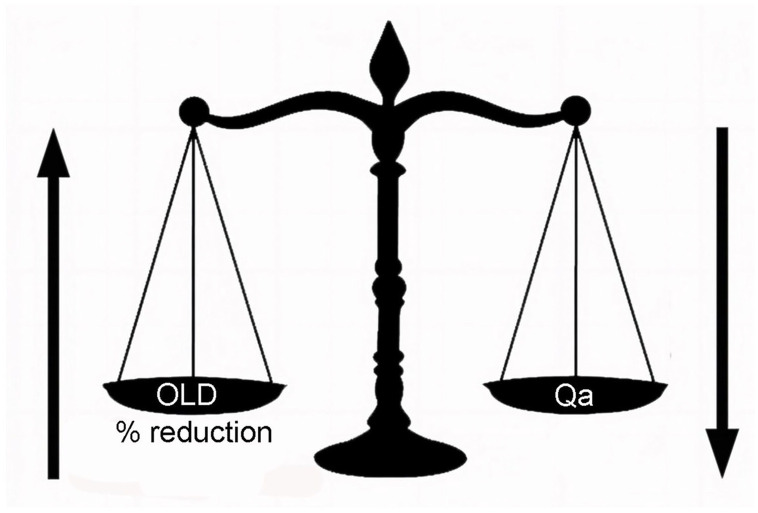
Reciprocal relationship between total cross-sectional outflow luminal diameter (OLD) and volume of inflow (Qa) in producing clinical indicators of imbalance.

These clinical indicators have a direct relationship to access blood flow (Qa) and pressure in the AV access. Although this situation is generally referred to as venous stenosis, a more accurate, descriptive, and clinically useful diagnosis, based upon pathophysiology, is inflow-outflow imbalance. In other words, the total outflow capacity of the AV access is inadequate to handle the inflow without an increase in pressure. The relative inadequacy of the access outflow capacity in comparison to inflow results in increased outflow resistance and a proportional increase in intraluminal pressure. Outflow capacity is often defined by a single enlarged venous outflow conduit. However, stenosis or occlusion of the main outflow vein may result in multiple collateral veins developing into adequate outflow capacity or in only partial outflow vein capacity improvement while remaining inadequate to normalize pressure and Qa. The clinical indicators associated with venous stenosis are the manifestations of this imbalance. The point at which this occurs is dependent upon variations in these two parameters—inflow volume and outflow resistance. The variations in these two parameters are considerable and reciprocal (Figure 1). Any given outflow luminal diameter (OLD) may not be problematic at a lower Qa but may be very problematic at a higher blood flow level or when increased due to arteriovenous anastomosis remodeling or percutaneous balloon angioplasty of a significant stenosis. In other words, as Qa increases, the total cross-sectional OLD required to create an increase in resistance decreases. With extremely high flow, the signs and symptoms of inflow-outflow embalance can be mainifest with no reduction in OLD. When clinically indicated, resolution of this common and potentially serious dilemma may be approached by one or both of two options: (1) reducing flow and pressure in the access or (2) increasing the OLD. In this review, we discuss such a bimodal approach to inflow-outflow imbalance.

## Treatment considerations

Most studies dealing with AV access Qa reduction have been for the treatment of an existing condition rather than its prevention; however, prevention of disease rather than waiting for its development is an important tenet of medical practice. With the ability to measure access flow easily and reliably, vascular access physicians have become more aware of the importance of the Qa component in the pathophysiology of this problem and the importance of intervention when indicated. Historically, our attention has been directed at prevention of thrombosis; however, this is only one of the many problems that can result from inflow-outflow imbalance. Excessive Qa results in or can lead to an entire list of serious problems that adversely affect patient morbidity and mortality (Table 2).

**Table 2.** Adverse effects of high access blood flow.

Eccentric left ventricular hypertrophy (LVH)
High output cardiac failure
Myocardial ischemia
Pulmonary hypertension
Peripheral and central venous stenosis
AV access-related hand ischemia
Decreased dialysis clearance secondary to high cardiopulmonary recirculation
Massively dilated fistula (megafistula)
Aneurysms and pseudoaneurysms
Inefficient dialysis

Patients may have venous outflow stenosis or occlusion yet have adequate outflow collaterals identified during routine examination or investigation for other problems. Such findings detected in asymptomatic patients with a normal access examination and acceptable Qa should be followed without intervention and with the expectation of continued successful vascular access performance. For patients with a high Qa or those who have clinical manifestations of high access pressure, two general approaches should be considered in dealing with these issues. First, in cases in which Qa is not excessive or is at least within a clinically acceptable range, treatment should be directed toward the anatomical lesion in order to reduce the pressure resulting from the outflow resistance. Second, in cases in which Qa is elevated, treatment should be directed toward the pathophysiology by reducing Qa to eliminate symptoms. In patients with a high flow access, treatment of the anatomical outflow lesion (if present) can serve to increase Qa even further, increasing the risks associated with elevated blood flow. In addition, since there is a direct correlation between Qa and both the development^2–6^ and recurrence of venous stenosis,^4,7^ Qa reduction can be expected to have a beneficial effect on the lesion involved. It is also important that Qa be evaluated after an angioplasty is performed. In some cases, treating a tight lesion can result in a significantly elevated Qa which would require flow reduction. All patients identified with access inflow-outflow imbalance, whether selected for observation or requiring intervention, should have planned follow-up as these conditions may evolve and recur over time.

## Anatomical indications for treatment

**Figure 2.**
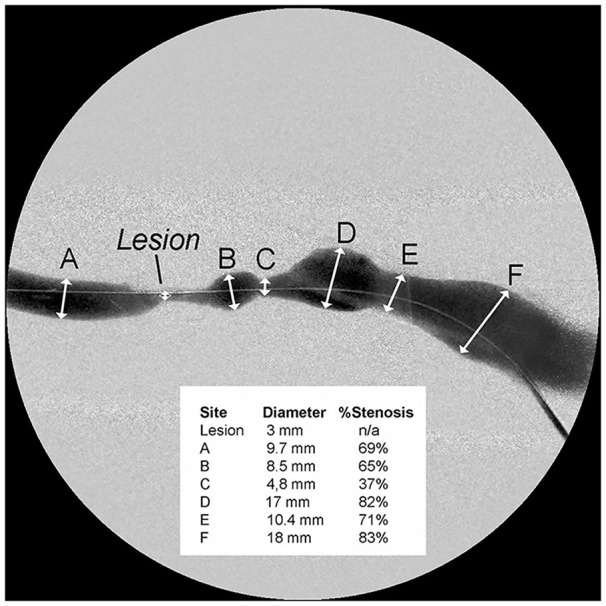
Variability in vein diameter.

The KDOQI Guidelines^1^ state that appropriate imaging of the dialysis circuit should identify the culprit lesion and suggest definitive treatment strategies. They further state that standard balloon angioplasty remains the treatment of choice for the majority of anatomic and clinically significant stenotic lesion. Recurrent lesions may require treatment with drug-coated balloons, covered stents, surgical bypass, or other interventions.^8^ A stenotic lesion that justifies treatment, referred to as critical stenosis, has been defined as one that is accompanied by clinical signs and symptoms (Table 1) and shows >50% narrowing relative to adjacent normal vein diameter by angiography or ultrasound.^1^ The 50% reduction in luminal diameter as a definition for critical stenosis is problematic for at least two reasons. First, the outflow vein is marked by varying degrees of diameter throughout its length,^9–11^ there is no adjacent normal vessel (Figure 2). When the area of narrowing is evaluated, the diameter proximally and distally often varies considerably.

### The effect of minimal lumen diameter

**Figure 3.**
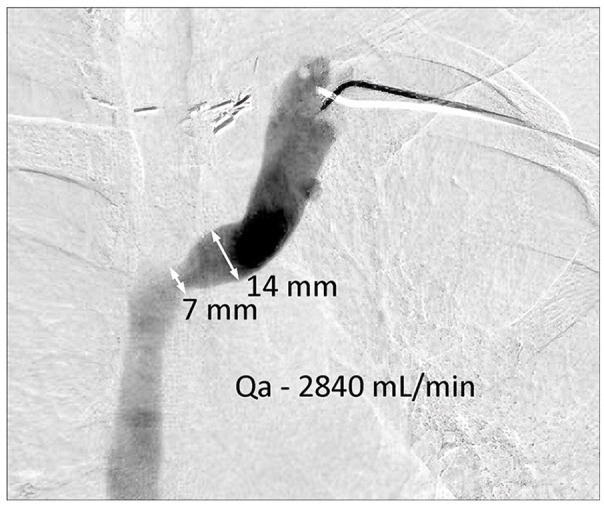
50% stenosis with a high Qa. The 7 mm segment in this two-dimensional image is not flow limiting.

Second, a 50% stenosis occurring in an AVF segment characterized by a diameter of 8 mm has very different flow characteristics from a 50% stenosis in a segment with a diameter of 14 mm. While both of these cases fit the definition of venous outflow stenoses, the pathophysiology as it relates to Qa is quite different. A major concern prompting the treatment of venous outflow stenosis is the concern that the lesion could result in a decreased Qa eventually leading to thrombosis of the AV access. However, the correlation between percentage stenosis and a clinically significant decrease in Qa is low.^10^ While a 50% decrease in luminal diameter in a 6 mm vein may produce a marked decrease in Qa, a 50% decrease in luminal diameter for a 14 mm vein may have no effect on flow or even be associated with an abnormally high Qa (Figure 3).

A stenotic lesion has a clinical effect because of its impact on blood flow. The term “critical stenosis” depends on hemodynamics associated with the lesion. Angiography alone cannot provide an estimate of the hemodynamic significance of a stenotic lesion.^12^ Computational fluid dynamics (CFD), based upon an arterial blood flow model,^12,13^ and combined clinical with CFD analysis based upon AVF models,^9,10,12,14^ have produced evidence that the hemodynamic changes associated with stenosis are not related to the percent decrease in luminal diameter but rather the absolute residual or minimal luminal diameter (MLD) and the length of the lesion. Clinical significance will be influenced by Qa and associated access pressure. Importantly, the determination of MLD can be more accurately accomplished because a comparator vessel is not involved. These factors favor this metric as a more reliable indicator for determining the significance of a stenotic lesion.

**Figure 4.**
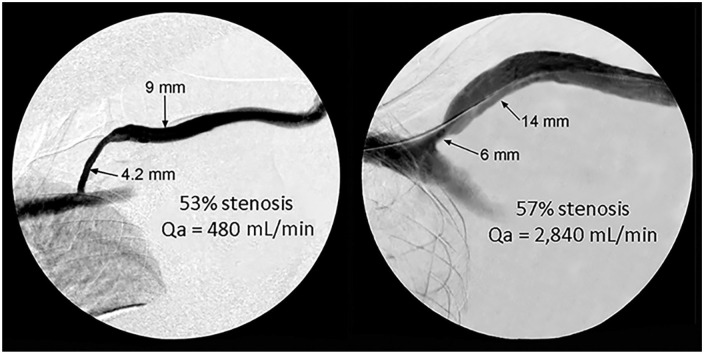
Comparison of two lesions with >50% stenosis but different Qa.

This principle is especially true when dealing with central venous lesions where the increased pressure resulting from a stenotic lesion can result in a markedly enlarged vein. While it may be impractical to measure MLD routinely, recognition of this principle is important, and the experienced operator can estimate MLD with a reasonable degree of accuracy.^10^ Central vein stenoses are not rare and many are asymptomatic, but they can become clinically manifest when Qa increases (arm swelling, increased venous pressure, prolonged bleeding after needle withdrawal among other problems). Dilating a venous segment to match an adjacent 12–14 mm vein is unnecessary and cannot be justified. The critical issue is the effect (if any) that the lesion has on Qa. Two lesions can appear similar based upon anatomical criteria but be quite different when Qa is considered (Figure 4). If Qa is not reduced, then the clinical problem relates to excessive inflow rather than restricted outflow. Importantly, balloon angioplasty treatment of such a non-critical venous outflow lesion not only carries the risk of associated procedural complications but may hasten progression of such lesions due to increasing neointimal hyperplasia.^15^

## Access blood flow indications for treatment

Increased blood flow rates through an AV access can lead to a host of problems such as cardiac disease (left ventricular hypertrophy, high-output cardiac failure, and myocardial ischemia), pulmonary hypertension, hemodialysis access-induced distal ischemia (HAIDI), aneurysm, and pseudoaneurysm formation^4–7,16–22^ (Table 2). The key to flow reduction treatment for excessive Qa lies in defining two issues: (1) the threshold Qa at which flow reduction should be performed, and (2) an optimal target Qa for the flow reduction procedure.

Although the exact threshold for defining a high blood flow AV access has not been rigorously validated nor universally accepted, a Qa of 1.5 L/min or >20% of the cardiac output (Qa/CO) are commonly used.^16^ It is clear that patients with a Qa >2000 mL/min are at increased risk of developing many complications including high-output cardiac failure.^23–25^ There are additional data to indicate that a Qa >1500 mL/min may well be associated with an increased cardiac risk.^18,26^ In addition, studies have shown that adverse changes in cardiac morphology occur relatively early after AV access creation and at lower blood flow rates.^21,23,24,27,28^ Based upon these considerations we suggest a threshold for AV access blood flow reduction of 1.5 L/min that is persistent^29^ after cardiopulmonary evaluation. Individuals with Qa >1500 mL/min are at increasing risk and should undergo a flow reduction procedure or be followed closely. However, it should be stressed that the increased Qa can also occur as a result of a higher level of hydration that increases the cardiac output via the for Frank-Starling law. Maintaining an appropriate dry weight setting is not easy and mild overhydration is common in patients treated by chronic hemodialysis. Therefore, the attempt to decrease dry weight should be done before any intervention for Qa reduction.

The second issue concerning the degree of flow reduction has been historically difficult with subjective banding procedures. Too much restriction resulted in thrombosis and too little restriction created no change in flow. This dilemma has been resolved with precision banding.^31^ K/DOQI Guidelines^41^ There are data to support the lower limit of Qa which will allow for effective dialysis and regional level of thrombosis avoidance. According to a Dialysis Outcomes Practice Patterns Study (DOPPS) survey^30^ dialysis facilities in the United States prescribed a mean blood flow rate (Qb) of 417 mL/min (median 400 mL/min). In general, the Qa should exceed this level by at least 100 mL/min to avoid excessive intra-access recirculation and thus gain facilitate effective dialysis. Studies have shown that a Qa of 500 mL/min defines a physiologically mature, clinically usable AVF.^31^ K/DOQI Guidelines^1^ report that those with an AVF, a Qa <500 mL/min or an arteriovenous graft (AVG) with <600 mL/min are at risk for the development of thrombosis. Thus, we feel that the target for flow reduction should generally be in the range of 600–800 mL/min. It should be noted that this level represents the value for cases in general. Individualization is important and requires good clinical judgment.

## Measuring access blood flow (Qa)

Access flow may be measured by a variety of means. Although an indicator dilution technique is more accurate,^32,33^ a common and reproducible technique is that of assessing mid-arm brachial artery flow by duplex ultrasound.^34^ The brachial artery possesses characteristics that make it an ideal surrogate for these measurements—laminar flow, a regular vascular wall with a circular cross-section area, and a remote probability of compression. In the setting of a flow reduction procedure, the brachial artery also provides a location remote from the operating site, avoiding inaccuracies resulting from operative intervention. Although this includes blood flow to the hand as well as to the AV access, the percentage of distal arterial flow is not enough to materially affect decisions related to management in most cases. However, in a branched outflow vein (AVF), brachial artery flow values may overestimate the “usable” flow for hemodialysis. For comparison, brachial artery blood flow in the resting arm in the absence of an arteriovenous dialysis access is in the range of 50–70 mL/min.^35,36^

This technique may be used in the clinic or at bedside and is a key element for a successful flow reduction procedure, ensuring proper restriction and procedure completion flow volume. Though not currently done routinely, the authors propose that measuring AV access flow in the interventional suite or procedure room before and after a flow reduction procedure is critical for success and allows precise adjustments to the restrictive technique (generally in 0.5 mm increments). Without such objective measurements, the planned flow reduction may be inadequate (no flow reduction) or too great (resulting AV access thrombosis). Another caveat that the proceduralist must be mindful of is that because there is a direct relationship between blood pressure and Qa, the effects of either an elevated blood pressure or a low blood pressure can be considerable and significantly affect the results obtained with flow reduction. A key element in achieving success with this procedure is maintaining the patient’s normal blood pressure during the flow restrictive procedure and Qa measurements. In order to do this, it is necessary to determine their normal baseline blood pressure. Patients undergoing an interventional procedure may experience an elevated blood pressure because of the associated stress and anxiety. In addition, the use of sedation/analgesia and de-hydration can result in a blood pressure below the patient’s normal level. In order to assure optimal results in these cases, intravenous medication, and hydration when appropriate, should be used to adjust the patient’s blood pressure to their normal baseline level, optimally a systolic pressure in the range of 130–140 mmHg.

## Flow reduction procedure

In the past, surgical banding has been used to address excessive AV access blood flow. This was often ineffective because it was not controlled by concurrent blood flow measurements and without objective measurement goals to predict success. As such, blood flow reduction was either insufficient or too excessive postoperatively, leading to ongoing symptoms of the condition being treated or to AV access thrombosis.^37–40^ The advent of precision banding^41–44^ has solved this problem.

**Figure 5.**
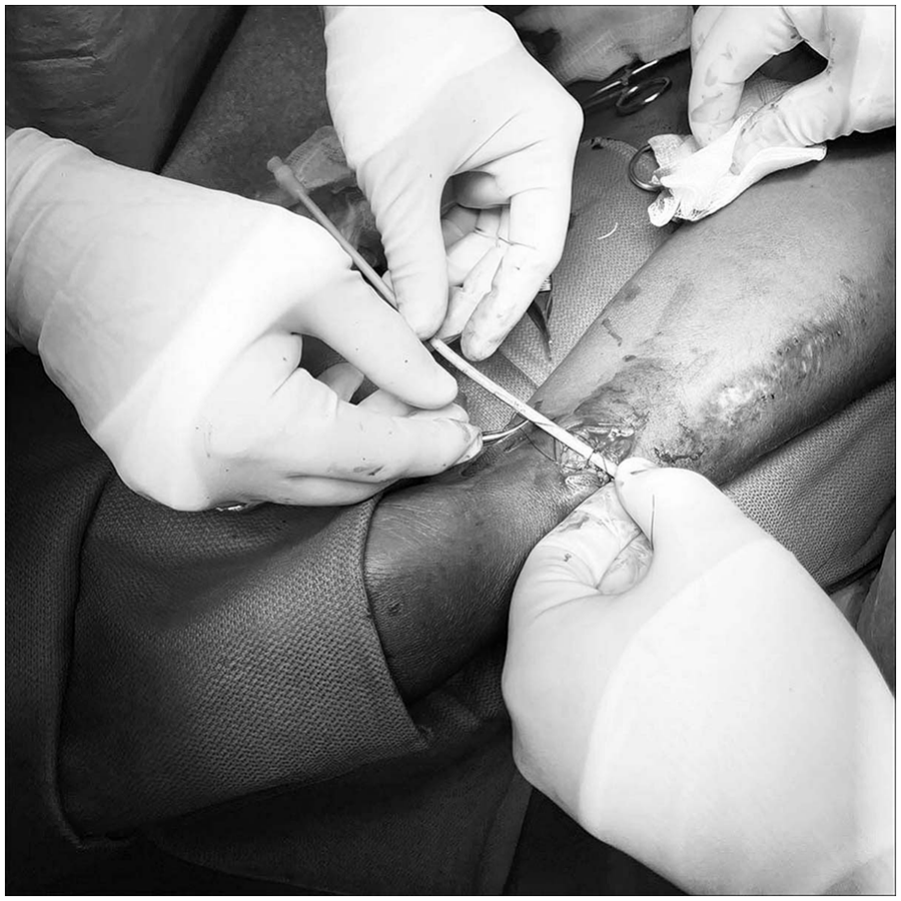
Endovascular, extraluminal precision banding using a 12 Fr dilator.

The precision banding technique is performed in conjunction with various techniques and materials for controlling the degree of lumen reduction together with the use of intraoperative ultrasound flow measurements before and after banding while adjusting the restriction in small increments.^42^ Common banding materials include permanent suture such as 2-0 polypropylene, 5–10 mm wide segments of polytetrafluorethylene fabric or a 5 mm band of polyester fiber suture material, among others. All of these items can be secured and adjusted to a precise restriction as indicated by flow measurements. Precise luminal restriction is achieved by using a sizing dowel of some type (often starting with a 12 Fr dilator, 4 mm coronary dilator, or 4 mm angioplasty balloon) placed either intraluminally or extraluminally, depending upon the device used.^42,45,46^ The combination of this technique for achieving luminal restriction guided by intraoperative blood flow measurements allows for precise adjustment of flow restriction outcomes.^47,48^ Post banding access flow may suggest a more or less restrictive band is needed to achieve the final Qa goal allowing additional adjustments to be made. Precision banding can be performed using either a surgical or an endovascular approach both of which are minimally invasive procedures.^39,42,49,50^ Although not appropriate for an AVG, endovascular precision banding for the treatment of a high blood flow AVF has become a common practice in many locations (Figure 5).

The largest report of precision banding involved a cohort of 297 patients with 398 encounters.^44^ Banding was performed using extraluminal endovascular technique. Median blood flow rate reduction in the series was 58%. Early banding failure occurred in 2.3% of patients, typically associated with an increase of the angiographic diameter at the banding site post-procedure, suggesting that the knots of the banding suture had slipped, suture had fractured, or the enfolded vessel wall had remodeled with wall thinning and loss of restriction. The 30-day thrombosis rate following banding was 3.8% with a median time to event of 5.5 days (2–102 days). The re-banding rate within a year was 14% with a median time to re-banding of 134 days (56–224 days).

There are cases in which a surgical approach has been recommended. Cases have been reported in which the AVF was markedly dilated, and suturing material was used for the banding, the suture eroded through a portion of the vessel wall and into the AVF outflow lumen.^51^ In addition, cases in which the banding was not immediately adjacent to the arterial anastomosis have resulted in pseudoaneurysm formation.^52^ As a result, it has been recommended that banding be placed as close to the arterial anastomosis as possible and if the AVF diameter exceeds 2 cm in the post-anastomotic segment, surgical revision of the anastomosis to a size smaller than the feeding artery be considered.^51,53^ Other surgical flow-reducing techniques include Revision Using Distal Inflow (RUDI), insertion of a narrower graft segment in the outflow conduit and extension of the access circuit, thereby adding an additional resistance component.^54,55^

### Bimodal approach to management

**Figure 6.**
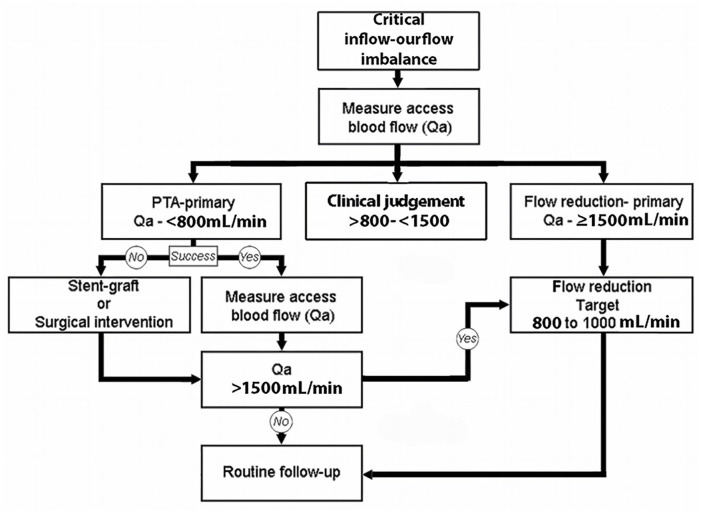
Algorithm for management of inflow-outflow imbalance, individualized treatment plans depending on clinical status. Symptomatic patients with lower flow volumes should have clinical evaluation to determine the potential benefit of inflow-outflow treatment options.

As part of the planning for the treatment of a patient who presents with clinical signs and symptoms suggesting the presence of critical venous outflow stenosis, Qa should be accessed pretreatment to identify and plan the appropriate treatment modality. Based upon measured Qa, we recommend that primary (initial) treatment for patients with a critical outflow stenosis should be categorized (depending on clinical evaluation) as either: (1) PTA-primary treatment—Qa of <600–800 mL/min for AVFs, or (2) flow reduction-primary treatment—Qa of >1500 mL/min for either an AVF or an AVG for a patient with a normal blood pressure (Figure 6). The rationale for these suggested threshold levels is derived from several factors. In most instances, a successful PTA will result in an increased Qa. Treatment of a lesion associated with a Qa >800 mL/min could potentially elevate AV access flow to a higher risk level, justifying the <800 mL/min upper limit for the PTA-primary treatment category. In the case of AV access flow >1500 mL/min, flow-reduction as primary treatment is suggested because if treated with PTA, a resulting increase in Qa could place it into a high flow risk category.

For cases with a Qa of 800–1500 mL/min, clinical judgment should be applied. In this category, AV access flow volumes should be interpreted in concert with careful medical history and physical examination in order to plan appropriate patient management. For an elderly, frail patient with reduced exercise tolerance, flow reduction should be considered. A patient with a distended pulsatile AVF, having prolonged post cannulation bleeding and a Qa of 900 mL/min, should benefit from PTA of an outflow lesion, although their posttreatment Qa will likely be increased, even moving into the need for flow restriction. A healthy subject having a Qa of 1200 mL/min with no evidence of problems related to excessive blood flow patient and an AVF that collapses with arm elevation may be best followed clinically with no intervention.

The authors propose that Qa be assessed post-treatment to determine if the increase in Qa is of such magnitude as to be of concern. PTA treatment of venous stenosis can result in an increase in Qa that may be anticipated if the AVF was hyper-pulsatile pretreatment. In some cases, the post PTA flow increase may be dramatic, resulting in emerging cardiopulmonary symptoms, hand ischemia, or arm edema. In these cases, the appropriate treatment is flow reduction. Long-term follow-up may be appropriate to monitor for later Qa elevation. Large studies involving precision banding have shown that recurrence of excessive AV access inflow can occur, particularly when post banding flow measurements were >1200 mL/min.^44^

## Summary

When a dialysis patient presents with clinical indicators suggestive of the presence of venous outflow stenosis, it is important to realize that these indicators are actually the result of an inflow-outflow imbalance. The treatment of this imbalance requires individualization based upon identification of the pathophysiology involved. A high flow AV access can have significant impact on the long-term morbidity and mortality of patients on dialysis, many of whom already have a considerable burden of comorbidities. A bimodal approach to therapy that includes an understanding and assessment of the underlying pathophysiology should be employed to optimize and individualize management. In many instances, angioplasty to treat the offending outflow lesion will be appropriate; however, in some cases, access flow reduction is required for management.
